# Rapid or Sudden Death in Infants from Unsuspected Causes

**Published:** 1899-07-22

**Authors:** Arthur Maude


### HOSPITAL CLINICS AND MEDICAL PROGRESS.

### RAPID OR SUDDEN DEATH IN INFANTS FROM UNSUSPECTED CAUSES.

By ARTHUR MAUDE, M.R.C.S., L.R.C.P.

SUDDEN death from unsuspected cause is by no means an uncommon event in infants under two years of age, and the difficulties in assigning the cause may be very great even at a post-mortem examination. It is of some interest, therefore, to general practitioners to consider the conditions which may lead to such a calamity, and the relative degree of frequency with which they may produce it.

From text-books of forensic medicine we should gather that all infants which die suddenly are suffocated either accidentally (by "over-lying") or by foul play; we need not consider, therefore, the evidence of such cases. But in ordinary practice by far the most frequent cause of rapid death is acute collapse of lung. In the infants of even well-to-do and careful parents, and in a far more frequent degree in those of the poor and neglectful, a broncho-pneumonia may be present for days, yet unsuspected; scarcely capable of producing noticeable ailment, but quite sufficient to produce a rapid and widespread collapse of lung, which may lead at any moment to cardiac failure or thrombosis of the right side of the heart. The condition is all the less noticeable and all the more liable to sudden fatal result when it occurs (as it so often does) in infants wasting from recent malassimilation, or in babies who have never been well nourished, and have consequently ill-developed and inadequate hearts.

Epidemic influenza is also a frequent cause of such disastrous rapidity of death. The initial symptoms in young infants are frequently very slight, and if not gastric or intestinal in type are often overlooked by parents, while in many cases the broncho-pneumonia produced is very rapid, and probably the specific organism expedites the end by affecting the cardiac nerve centres. The next place in production of rapid death is taken by laryngismus stridulus, spasm of the glottis, unassociated with laryngitis, and almost invariably occurring in ricketty children.

"Sudden death from asphyxia may take place early, even, it is said, in the first attack,"[1] and no appearance may be present at an autopsy beyond those of early rickets, coupled with evidence of asphyxia. Pure uncomplicated laryngismus may cause sudden death, but an additional danger is now recognised in incarceration of the epiglottis produced by a violent paroxysm. In all cases of sudden asphyxia the pharynx should be explored with the finger to ascertain if this accident has taken place, and to release the epiglottis if the child is still alive.

Laryngismus in new-born babies is very apt to be fatal in an early attack, for it usually occurs in weakly or premature infants; and the attacks of asphyxia, though short, are ill-borne, and may produce rapid and increasing collapse of lung, which in turn accentuates the dangers of a first attack; while in new-born infants the very feebleness of the child renders the signs of the initial paroxysms less noticeable, so that an apparently "sudden" death may follow on attacks which have been present, but have escaped observation even of fairly careful persons.

Next we must bear in mind that young infants frequently die of convulsions during a convulsion, no matter what the cause of the convulsion may be. Elder children and adults hardly ever die during a convulsion,[2] if we except the prolonged convulsions of a "status epilepticus" and such rare conditions as hydrophobia and tetanus. But not only do young babies die in convulsions, but the first attack of convulsions may be very severe, and they may die not only in it, but early in it, in the first few seconds of attack, so that death may occur in a very sudden and inexplicable manner. Tetanus neonatorum—trismus neonatorum as it used to be called—is a condition seldom seen in England, or at least recognised; but it was apparently common here in the seventeenth century, if we accept Dr. Gee's[3] scholarly interpretation of Willis's description; while it is common still in the cotton plantations of the Southern States, and in the West Indies, and in St. Kilda and Scotland, in all such places where the umbilicus of the infant may be readily infected from manured soil by the dirty hands of midwives who toil in fields and gardens. In the poorer parts of London, moreover, many infants perish from "convulsions" a few days after birth; in all such cases a strong suspicion of tetanus must exist, and the possibility of infection from manure of mews or gardens should be investigated and, if possible, cultivations be taken by a bacteriologist.

Another very possible cause of rapid death is diphtheria. In feeble infants of a few weeks old the signs of the throat affection may quite escape the observation of careless or negligent parents, while even among the children of the well-to-do the initial throat trouble may be so slight as to postpone appeal to professional advice until thrombosis of the right side of the heart or cardiac failure from diphtherial intoxication has terminated fatally. In either case death may be apparently very sudden, though had the child been seen during life the second condition would have afforded evidence from the character of the pulse.

The rarer conditions are of great interest. Hæmorrhage into the supra-renal capsules is a frequent result of traumatism during birth. In 130 stillborn children Dr. Herbert Spencer[4] found extravasations into the capsules present in 26. They occur in any difficult or prolonged labour, but are more common in pelvic presentations. There is as yet no evidence when life is possible for any time after such an accident, or if the remaining gland is capable of carrying on the work of both.

Dr. Buzzard has suggested that sudden death in young children may be sometimes due to infantile paralysis affecting the medulla with the same abruptness that characterises its invasion of the anterior cornua. This is scarcely yet more than an hypothesis, but acquires confirmation from the accumulating evidence that acute anterior poliomyelitis is an infective process; for according to Schultze[5] the combination of localised meningitis with cervical poliomyelitis is by no means rare, the relations resembling those of an infective pleurisy and pneumonia, and in at least one instance the Weichselbaum-Jaeger diplococcus has been discovered in spinal fluid removed by a Quincke's puncture.

Intracerebral hæmorrhage in infants is rare,[6] but hæmorrhage into the arachnoid sac is by no means unknown in prolonged or instrumental labour, and may cause death a few days after birth.

Lastly, infants having general tuberculosis not infrequently die with startling rapidity, death being heralded by no symptoms apart from the general chronic ones, save a sudden, sharp rise of temperature.

[1] Eustace Smith, Disease in Children, 1884, p. 271. [2] West, Diseases of Infancy, 1874, p. 187. [3] St. Bart. Hosp. Rep., 1867, p. 101. [4] Obstet. Soc. Trans., 1892; Allbutt's Syst. Med., vol. 4, p. 568. [5] Munchener Med. Woch., Sept. 20, 1898. [6] Eustace Smith, Op. cit.

